# Analysis of Body Composition and Levels of Antimicrobial Peptides in Patients with Basal Cell Carcinoma: A Preliminary Study

**DOI:** 10.3390/jcm14020419

**Published:** 2025-01-10

**Authors:** Marta Fijałkowska, Bogusław Antoszewski, Mateusz Koziej

**Affiliations:** 1 Department of Plastic, Reconstructive and Aesthetic Surgery, Medical University of Lodz, 90-153 Lodz, Poland; 2 Department of Anatomy, Jagiellonian University Medical College, 33-332 Cracow, Poland

**Keywords:** skin, basal cell carcinoma, body composition, cathelicidin, defensin, visceral fat

## Abstract

**Background**: Excessive body fatness is the basis of many diseases, especially civilization-related ones. The aim of this study is to analyze the body composition and serum levels of selected antimicrobial peptides (AMPs) in patients with basal cell carcinoma (BCC), in comparison to healthy controls (HCs), and investigate whether any specific parameter significantly increases the risk of BCC development. **Methods**: The body composition and measurements of serum levels of cathelicidin and human-beta-defensin-2 were analyzed in a group of 100 subjects (50 patients with BCC and 50 HCs). **Results**: There were statistically significant differences between the visceral fat rating (BCC 11.7 vs. control 10.1), cathelicidin (BCC 1022.6 vs. control 428.4), defensin-2 (BCC 1.2 vs. control 0.4), age (BCC 68.7 vs. control 62.4), and the visceral fat/muscle ratio (BCC 0.24 vs. control 0.21). **Conclusions:** It seems that excessive fat, especially visceral fat, may pose a risk of developing skin cancer. Therefore, it should be taken into account when caring for patients and they should be made aware that losing body weight may be important not only in reducing the risk of hypertension or diabetes but also cancer diseases. There are numerous well-known risk factors for developing skin cancer, but few are modifiable. Among these modifiable factors is the patient’s weight and body composition, so improvaing lifestyle is crucial in the prevention of skin cancers.

## 1. Introduction

Body Mass Index (BMI) is a widely used parameter to determine whether a patient has a normal body weight, is overweight, or is obese. However, though BMI testing provides a reasonably accurate measure of weight and health, it offers limited additional insight as it does not provide an accurate measure of body composition or body fat distribution. More comprehensive information can be obtained by monitoring human body composition. Body composition measurements typically rely on bioelectrical impedance, allowing for the assessment of total body water (TBW), fat-free mass (FFM), fat mass, muscle mass, bone mass, and visceral fat rating (VFR) [1,2,3]. There is also a possibility to use imaging techniques like computed tomography (CT) or magnetic resonance imaging (MRI). By using CT or MRI, the depots of subcutaneous and visceral adipose tissue can be quantified and differentiated. Nevertheless, it is time-consuming, the cost is quite high, and there is a need for radiation usage in the case of CT, so these techniques are not very useful in everyday clinical practice [2]. That is why bioelectrical impedance seems to be an accessible, safe, and cost-efficient alternative method that has been widely used to measure body composition in clinical studies [2].

During the past decades, obesity has become one of the leading causes of death and disability. The latest data show that more than one-third of Americans and about one-fifth of Europeans are obese [2]. This condition is associated with the development of many diseases like cardiovascular disease, hypertension, diabetes mellitus, dyslipidemia, stroke, and cancers [2]. Obesity is a complex, multifactorial condition characterized by overaccumulation of body fat, especially in the area of the abdomen. Adipose tissue is recognized as an extremely active endocrine organ. In recent years, it has been explained that adipose tissue secretes a wide variety of molecules involved in the control of many physiological processes such as the regulation of appetite, body weight, growth, reproduction, and immunity, among others [2]. Not only is the amount of adipose tissue essential, but its distribution also plays an important role in the incidence of obesity-associated comorbidities, with visceral depot showing a more pathogenic profile than subcutaneous fat [2]. The involvement of VFR in obesity-associated morbidity and mortality is increasing.

The analysis of body composition is an important and helpful aspect of diagnosing and treating diseases such as obesity, cardiovascular problems, chronic liver illnesses, or inflammatory bowel disease [1,4,5,6]. In the literature, there are papers describing the correlation between increased body fat and various types of cancers [7,8,9]. The International Agency for Research on Cancer in 2016 found that there is evidence that excess body fatness can be associated with an increased risk of at least 13 different types of cancers (endometrial, postmenopausal breast, colorectal, esophageal, renal/kidneys, meningioma, pancreatic, gastric cardia, liver, multiple myeloma, ovarian, gallbladder, and thyroid) [10]. There are a few hypotheses attempting to explain the association between body composition and cancer. One argument suggests a correlation between increased height or weight and the total number of cells that might undergo malignant transformation [11]. Another suggests a direct connection between adult cancer incidence and caloric intake in early life [11]. In terms of the carcinogenesis mechanisms of obesity, it has been proposed that adipose tissue secretes several cytokines and hormones believed to promote inflammation, cell proliferation, and angiogenesis, thereby promoting tumor growth [12]. Changes in metabolic functions and sex-hormone-level disorders also are likely mechanisms linking excess body fat to cancer formation [13]. Anabolic endocrine hormones like insulin and insulin-like growth factor 1 (IGF-1) play an important role in glucose metabolism, angiogenesis, cell proliferation, and death [13]. Additionally, excess body fat, particularly visceral fat, is positively correlated with insulin resistance. When there are permanent high levels of blood glucose, excess insulin is secreted from the pancreas which results in hyperinsulinemia leading to increased levels of free IGF-1—this may promote carcinogenesis [14]. In women, especially after menopause, the pathways described above also modify the bioavailability of sex hormones. Prolonged hyperinsulinemia reduces bioavailable sex hormone-binding globulin (SHBG) and increases circulating estrogens and androgens, which may further promote carcinogenesis [14].

The role of the immune system in carcinogenesis is unquestionable, and recent studies have demonstrated that small endogenous antimicrobial peptides (AMPs) (such as cathelicidin and defensins) play a significant role in eliminating tumor cells [15,16,17]. In our previous study, we found that serum levels of cathelicidin and human-beta-defensin-2 (HBD-2) are more elevated in patients with basal cell carcinoma (BCC) compared to healthy controls [18]. Gambini et al. paid attention to the fact that dysregulation of the local immune environment plays a remarkable role in BCC formation [19]. Recently, the main interest in the pathogenesis of BCC has been in the tumor microenvironment (TME). The most vital immune cells in the TME are the so-called tumor-infiltrating lymphocytes (TILs), of which regulatory T-cells (T-regs) seem to be the most important as they demonstrate immunosuppressive activity. Studies on BCC have shown the presence of a high concentration of T-regs, not only in the TME but also in the tumor-bordering skin, which can be chemoattracted by different chemokines [20]. TILs in BCC seem to be recruited from circulating cell pools rather than from resident T-cells [21].

Consequently, KC is the most frequently occurring cancer worldwide, with BCC being the most common type found in humans. There is value in determining whether there are any body composition variables in patients with BCC that can be linked to the occurrence of skin tumors. Additionally, it would be intriguing to investigate whether weight gain influences serum AMP levels, given that an increase in adipocyte volume often leads to a higher number of immune cells accumulating in fat tissue. This accumulation subsequently generates a pro-inflammatory cytokine and hormonal environment [19].

This paper aims to analyze the body composition and serum levels of selected AMPs in patients with BCC, comparing them to healthy controls. The goal is to identify which parameters significantly increase the risk of BCC development.

## 2. Materials and Methods

The study group consisted of 100 consecutively admitted patients treated in our department: 50 patients underwent operations for skin lesions suspected to be basal cell carcinoma, all of whom were non-smokers, their oncological history was negative, and they did not have other autoimmune diseases. The other 50 patients underwent surgery for various other medical concerns, primarily aesthetic ones, and were admitted for one-day procedures. Control patients were also non-smokers and free from any malignancies or autoimmune diseases. Of all examined patients, 68 had hypertension, and 12 had diabetes mellitus type 2 (therefore, the pharmacological anamnesis included medications for high blood pressure and diabetes). Additionally, each participant was asked about the presence of cancer in the family; in all cases, familial history was negative. All 50 patients with skin neoplasms underwent their operations under local anesthesia, and the excised skin tumors were sent for histopathological examination. The results, obtained after 2 weeks, showed that all these patients were diagnosed with BCC.

Before surgery, as standard, all subjects had an intravenous (IV) cannula inserted, and this IV line was used to collect a 10 mL blood sample. Immediately after collection, the blood was delivered to our hospital laboratory and centrifuged to obtain plasma. Each sample was then frozen and stored at a temperature of minus 80 degrees Celsius until analysis. After all 100 samples were collected, the plasma was refrozen. The concentrations of beta-defensin-2 (HBD-2) and cathelicidin were then measured using an Elisa Kit (Wuhan EIAab Science Co., Ltd, Biopark, Optics Valley, Wuhan, China, 430074).

The anthropometric measurements—body composition and height—were performed on all examined patients just before surgery using a body composition analyzer (Tanita BC-420 MA). The equipment was always located in the same place in the nurses’ preparation room and was properly leveled. Patients were asked to remove metal items, such as belts, earrings, and necklaces, as well as their outerwear. Examinations were conducted with patients in a standing, barefoot position, with feet placed on metal platforms as indicated by the supervisor. Height was measured using an anthropometer. Body height is the distance measured from the base to the highest anatomical point on the head (vertex). When taking the measurement, the participant stood with their back to the height meter in an upright position. The correct position was when the head, shoulders, buttocks, and heels touched the anthropometer. The arms were placed along the body, and the head was positioned so that the ear canal was in line with the cheekbone. Height was measured without shoes, hats, or hair ornaments. Performed measurements were non-invasive, the proper information concerning body type, sex, age, and height was introduced by the doctor followed by machine instructions, and the results were printed directly and collected with patients’ files.

All patients signed written consent forms prior to enrollment in the study. The project was approved by the Ethics Review Board of the Medical University of Lodz (approval no. RNN/364/18/KE), and the study was conducted following the principles of the Declaration of Helsinki.

### Statistical Analysis

Data are presented as mean values with corresponding standard deviations. StatSoft Statistica 13 for Windows was used for all statistical analyses. *p*-values under 0.05 were deemed statistically significant. Correlation coefficients were established to measure statistical dependence, while Student’s *t*-tests and Mann–Whitney U-tests were conducted for statistical comparisons.

## 3. Results

The average age for the study group was 65.5 ± 11.2. For the BCC group, the average age was 62.4 ± 10.1, while it was 68.7 ± 11.4 for the control group. The BCC group consisted of 19 men (38.0%) and 31 women (62.0%). On the other hand, the control group consisted of 14 men (28.0%) and 36 women (72.0%).

The mean values along with standard deviations of the obtained measurements and calculations are presented in Table 1. There was a statistically significant difference between the VFR (BCC 11.7 ± 3.7 vs. control 10.1 ± 4.0), cathelicidin (BCC 1022.6 ± 1259.9 vs. control 428.4 ± 187.5), defensin-2 (BCC 1.2 ± 1.6 vs. control 0.4 ± 0.2), and age (BCC 68.7 ± 11.4 vs. control 62.4 ± 10.1), as well as the visceral fat/muscle ratio (BCC 0.24 ± 0.06 vs. control 0.21 ± 0.07). All of these values were higher in the BCC group. It is also noteworthy that the majority of the control group subjects (38 individuals, representing 76%) reported gaining weight (5 kg or more) over the past 3 months, whereas 42 BCC patients (84%) stated their weight remained stable during the same period (*p* > 0.05).

The associations between measured parameters and the biochemical parameters cathelicidin and defensin-2 are demonstrated in Table 2. In the BCC group, only age was inversely correlated with defensin-2 (R = −0.3; *p* = 0.036). Conversely, in the control group, parameters such as age, weight, height, BMI, fat mass, muscle mass, bone mass, TBW, VFR, obesity level percentage, and the visceral fat/muscle ratio were correlated with either cathelicidin or defensin-2.

## 4. Discussion

The number of studies describing the relationship between KC and BMI is limited, and the results are not clear. An analysis performed by Pothiawala et al. revealed that obesity appears to be inversely associated with the development of BCC and SCC for both women and men [12]. Chan et al. suggested that not only do obese women demonstrate a lower hazard rate of KC obese women but so do overweight women (BMI > 25 kg/m^2^) [22]. Præstegaard et al. found that elevated BMIs decreased the risk of BCC in women, while no such link was found for men [23]. A study conducted by Cai et al. found no evident association between BMI and risk of KC [24]. Olsen et al. also did not identify any connection between BMI and the occurrence of BCC, yet they highlighted that weight gain amongst women slightly increased the risk of skin cancer, though not to a statistically significant level [11].

In our analysis, there was no link between the occurrence of BCC and BMI. However, the VFR was significantly higher in patients with BCC compared to healthy controls. Fat distribution in the human body and its excess level determines the type of obesity (general, abdominal, or gluteofemoral). Even though visceral fat deposit (about 20% of total body fat) is much smaller than peripheral (subcutaneous) adipose tissue (about 80% of total body fat), it shows a remarkable association with serious health issues [25,26,27]. Visceral fat ratings from 1 to 12 indicate that a patient has a healthy level of visceral fat, while ratings above 13 suggest an excess level of visceral fat [27]. In our groups, both BCC patients and healthy controls had VFRs lower than 12. However, BCC subjects were close to crossing the threshold of 12 (Table 1).

It is well known that visceral fat plays a critical role in releasing various pro-inflammatory cytokines, leading to chronic inflammation due to the immune activity of adipose tissue [25]. Small AMPs can also be released by adipose tissue, and the levels of cathelicidin and HBD-2 may be elevated in patients with BCC, as demonstrated in our previous study [18]. Interestingly, this analysis showed that the levels of cathelicidin and defensin-2 were significantly higher in the BCC group than in the control group (Table 1). Moreover, we found significant correlations between the level of selected AMPs and body composition parameters, favoring subjects from the control group over those from the BCC group (Table 2), which can be related to weight gain reported by healthy controls.

Research exploring the associations between AMP levels and body composition is limited. Benachour et al. investigated the relationship between cathelicidin, BMI, and the risk of cardiovascular disease [28]. Their results indicated a significant positive correlation between cathelicidin expression and BMI [28]. Kozłowska et al. found that cathelicidin levels are higher in women with depression, and this level is also positively related to the mass of visceral adipose tissue, but not BMI [29]. However, similar studies concerning HBD-2 and body composition are lacking.

In our study, we identified significant correlations between cathelicidin, defensin-2, and various body composition parameters, favoring healthy subjects. Interestingly, this can potentially be explained by the weight gain reported by healthy control patients in the last 3 months, as opposed to the BCC patients. As proposed by Szczepocka et al., an increase in AMPs in situations such as obesity is expected, as cathelicidin is linked with inflammatory diseases, and the mechanism connecting obesity with altered antimicrobial peptide expression may be located within the adipose tissue [30]. It is worth noting that not only is obesity a health risk but so is being overweight, with the secretory activity of adipose tissue making weight gain a critical parameter.

It can be expected that, by having a positive association between obesity and/or weight gain and cancer, weight loss may be a practicable prevention approach for reducing the risk of cancer. As it was shown by Friedenreich et al. in their systematic review that included 34 studies, 16 revealed a statistically significant reduction in the risk of cancer in individuals who experienced weight loss [13]. What was also shown in our study was the fact that weight gain can elevate the level of AMPs in patients without skin cancer; this may, indirectly, prove that increased adipocyte activity modulates the immune system causing chronic inflammation. Since there are many factors responsible for skin cancer development, it is important to focus on those factors that are modifiable, and one of them is body weight.

To our knowledge, this is the first study comparing the body composition and levels of selected AMPs in patients with BCC and healthy controls. Other papers primarily described only BMI, and while this indicator has some clinical significance, it does not provide information about the type of obesity. We measured body composition and found that visceral obesity is a significant factor in increasing the risk of BCC occurrence. These measurements were obtained prospectively by doctors using standardized methods, thereby minimizing the possibility of differential recall bias.

## 5. Conclusions

It seems that excessive fat, especially visceral fat, may pose a risk of developing skin cancer. Therefore, it should be taken into account when caring for patients and they should be made aware that losing body weight may be important not only in reducing the risk of hypertension or diabetes but also cancer diseases.

In the future, if the analyzer is available, it is worth considering body composition analysis for each patient admitted to a clinic or hospital. This will allow us to estimate the scale of overweight and obesity in the examined population and, combined with medical and family history, to assess the risk of developing lifestyle diseases, including skin cancer. Such a test is quick and non-invasive and can be used as a standard screening method.

This study is not free from limitations. First, the number of subjects was limited, suggesting the need for further studies on body composition in patients with BCC. Second, our analysis only included patients with BCC; those with squamous cell carcinoma were not part of the study. Additionally, while our focus was on body composition and the levels of cathelicidin and HBD-2, we did not consider other risk factors such as UV radiation exposure, sunburns, ionizing radiation exposure, and pigmentation of skin, hair, and eyes in the current analysis. Further studies combining measurements of all mentioned variables may allow for the creation of a predictive model for assessing the risk of skin cancer in a particular patient.

## Figures and Tables

**Table 1:** Comparison between measured parameters between patients with basal cell carcinoma (BCC) and control group.

	BCC		Control		*p*
	Mean	SD	Mean	SD	
Weight [kg]	75.4	13.2	78.2	20.6	0.825
Height [cm]	165.0	8.6	167.3	9.6	0.365
BMI	27.8	4.1	27.2	4.6	0.321
% of fat	32.5	8.1	32.3	9.1	0.956
Fat mass	25.0	8.4	25.0	10.2	0.899
Muscle mass	48.3	9.5	48.8	11.2	0.909
Bone mass	2.6	0.5	2.6	0.6	1.000
TBW [kg]	1023.7	6989.9	35.5	8.0	0.890
TBW [%]	46.4	5.1	46.7	5.4	0.804
Ideal mass	60.1	6.4	61.8	7.3	0.347
Visceral fat rating	11.7	3.7	10.1	4.0	0.035
Obesity level in %	26.4	18.5	23.4	21.1	0.292
Cathelicidin	1022.6	1259.9	428.4	187.5	0.026
Defensin-1	5.6	17.8	4.3	15.2	0.866
Defensin-2	1.2	1.6	0.4	0.2	0.036
Age	68.7	11.4	62.4	10.1	0.004
Fat/muscle	0.53	0.19	0.53	0.22	0.926
Visceral fat/muscle	0.24	0.06	0.21	0.07	0.005

**Table 2:** Associations between cathelicidin, defensin-2, and other body parameters in patients with basal cell carcinoma (BCC) and control group.

	BCC				Control			
	Cathelicidin		Defensin-2		Cathelicidin		Defensin-2	
	R	*p*	R	*p*	R	*p*	R	*p*
Age	−0.14	0.378	−0.30	0.036	0.33	0.037	−0.18	0.203
Weight [kg]	0.09	0.579	−0.08	0.558	0.68	0.000	0.65	0.000
Height [cm]	0.25	0.113	0.24	0.098	0.14	0.390	0.28	0.049
BMI	−0.11	0.506	−0.22	0.131	0.81	0.000	0.65	0.000
% of fat	−0.19	0.236	−0.25	0.075	0.19	0.224	0.21	0.152
Fat mass	−0.09	0.589	−0.19	0.193	0.44	0.004	0.45	0.001
Muscle mass	0.17	0.290	0.08	0.569	0.51	0.001	0.55	0.000
Bone mass	0.18	0.271	0.07	0.615	0.49	0.001	0.53	0.000
TBW [kg]	0.15	0.361	0.08	0.560	0.52	0.000	0.58	0.000
TBW [%]	0.15	0.336	0.20	0.154	−0.19	0.235	−0.16	0.270
Ideal mass	0.25	0.114	0.24	0.099	0.14	0.371	0.28	0.050
Visceral fat rating	0.08	0.621	−0.16	0.258	0.68	0.000	0.34	0.015
Obesity level in %	−0.10	0.516	−0.22	0.134	0.80	0.000	0.64	0.000
Fat/muscle	−0.18	0.258	−0.24	0.086	0.19	0.231	0.20	0.160
Visceral fat/muscle	−0.01	0.962	−0.25	0.082	0.51	0.001	0.12	0.423

## Data Availability

Data can be obtained from the corresponding author after an appropriate request.
